# Metalearning approach for leukemia informative genes prioritization

**DOI:** 10.1515/jib-2019-0069

**Published:** 2020-05-08

**Authors:** Vânia Rodrigues, Sérgio Deusdado

**Affiliations:** USAL – Universidad de Salamanca, 37008, Salamanca, Spain; CIMO – Centro de Investigação de Montanha, Instituto Politécnico de Bragança, 5301-855, Bragança, Portugal

**Keywords:** informative genes, leukemia, machine learning, metalearning, microarray

## Abstract

The discovery of diagnostic or prognostic biomarkers is fundamental to optimize therapeutics for patients. By enhancing the interpretability of the prediction model, this work is aimed to optimize Leukemia diagnosis while retaining a high-performance evaluation in the identification of informative genes. For this purpose, we used an optimal parameterization of Kernel Logistic Regression method on Leukemia microarray gene expression data classification, applying metalearners to select attributes, reducing the data dimensionality before passing it to the classifier. Pearson correlation and chi-squared statistic were the attribute evaluators applied on metalearners, having information gain as single-attribute evaluator. The implemented models relied on 10-fold cross-validation. The metalearners approach identified 12 common genes, with highest average merit of 0.999. The practical work was developed using the public datamining software WEKA.

## 1 Introduction

The type of leukemia is determined by the stage of development of the cell when it becomes malignant or cancerous. Acute lymphoblastic leukemia (ALL) is the most common type of leukemia in childhood, targeting the lymphoid line of blood cells [1]. Acute myeloid leukemia (AML) affects the myeloid line of blood cells and is a fast-growing form of cancer of the blood and bone marrow.

The occurrence of cancer or subtype cancer can be determined through the informative genes, considering pattern expressions and its correlation to cancer typology. For this purpose, statistical methods and machine learning techniques can be employed for feature selection and, in this way, prioritizing informative genes.

The objective of this work was to identify an optimal subset of genes as best diagnostic markers for leukemia, inferred from the best results from performance evaluation in classification implementing Kernel Logistic Regression (KLR). KLR model is a statistical classifier [2] that generates a fit model by minimizing the negative log-likelihood with a quadratic penalty using the Broyden–Fletcher–Goldfard-Shanno (BFGS) optimization [3].

Machine learning tools and techniques allow the implementation of metalearners. Metalearning algorithms use classifiers as powerful learners. An attribute selection classifier is a metalearner example. It contains parameters such as filter and search method, which allow to reduce dimensionality of data by attribute selection, without loss information [4].

Filter methods are one of the three general classes of feature selection algorithms. They apply a statistical measure to assign a scoring to each feature. The features are ranked by its score and accordingly selected to be kept or removed from the dataset. The methods are often univariate and consider the feature independently, or with regard to the dependent variable. Examples include chi-square [4], correlation coefficient [5], and information gain [6].

This paper has been structured as follows. After a brief introduction, in Section 2 we explain the methodology followed in this study, as well as the procedures, concluding with the performance assessment of the classification methods. Details of the experimental work using WEKA datamining workbench, plus the obtained results are discussed in Section 3. The conclusions are presented in Section 4.

## 2 Methods

### 2.1 Experimental procedures

**Figure 1:**
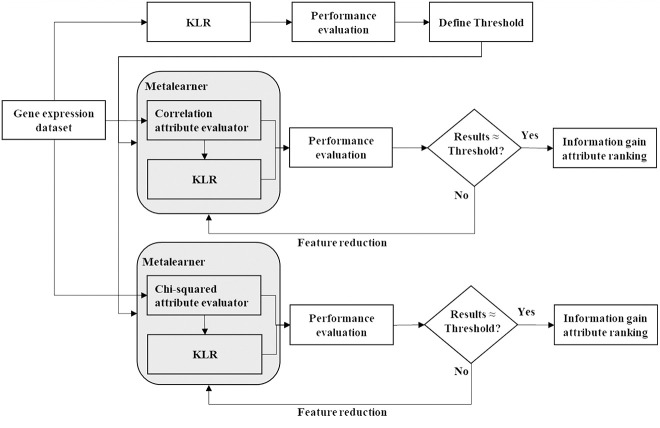
Procedures workflow.

The experimental work was based on the WEKA, version 3.8.3, a datamining workbench publicly accessible at: www.cs.waikato.ac.nz/ml/weka/. In this work, two metalearners were applied to reduce dimensionality of data by attribute selection. The procedures workflow is shown in Figure 1. Correlation attribute evaluator and chi-squared attribute evaluator were chosen as supervised filter methods before being passed by KLR. The optimal parameterizations of KLR were described in Refs. [7]. These experiments ran 10 times several schemes with 10-fold cross-validation testing with Paired T-Tester (corrected). The number of attributes to retain was chosen after several tests and validating the results of performance evaluation through comparison with results obtained when the classifier was applied on the original number of attributes. After, information gain was applied on the attributes retained by the two metalearners and the rank proceeded according to their evaluation. Moreover, biological interpretation of the subset of genes selected was extracted from literature. These set of experiments were conducted on a computer with an Intel Core i7-5500U CPU 2.40 GHz processor, with 8.00 GB RAM.

### 2.2 Datasets

The Leukemia dataset was obtained online from http://portals.broadinstitute.org/cgi-bin/cancer/publications/pub_paper.cgi?mode=view&paper_id=63, and was published as part of the experimental work in Refs. [8]. It includes two types of leukemia: ALL and AML. The dataset was analyzed in a reduced version, composed by 28 samples keeping the same features (12,582 genes). The goal for this subdivision was to identify informative genes in balanced data.

### 2.3 Performance evaluation

We have trained the classifiers to predict outcomes of cancer microarray datasets containing positive samples and control samples as described in Refs. [7]. The evaluation measures to evaluate the classifiers [9], [10] includes classification accuracy (*ACC*), i. e., the ratio of the true positives and true negatives obtained by the classifier over the total number of instances in the test dataset, defined as:

$$ACC=\frac{TN+TP}{TP+FP+FN+TN}$$

Kappa (*κ*) coefficient is a statistical measure for qualitative (categorical) items as given by:

$$\kappa =\frac{Observed\;Accuracy-Expected\;Accuracy}{1-Expected\;Accuracy}$$

Kappa coefficient is interpreted using the guidelines outlined by Landis and Koch (1977), where strength of the *κ* is interpreted in the flowing manner: 0.01–0.20 slight; 0.21–0.40 fair; 0.41–0.60 moderate; 0.61–0.80 substantial; 0.81–1.00 almost perfect [11].

Mean absolute error (MAE) measures the average magnitude of the errors in a set of prediction, without considering their direction [12]. It is given by:

$$MAE=\frac{\sum_{i=1}^{n}\left|predicted_{i}-{\text{actual}}_{i}\right|}{total\;predictions}$$

Precision (*PRE*), it is also called the Positive predictive values (PPV), is the proportion of the true positives against the true positives and false positives, as given by equation:

$$PRE=\frac{TP}{TP+FP}$$

Recall (*REC*) also called sensitivity and hit rate, is the proportion of the true positives against true positives and false negatives, as given by the equation:

$$REC=\frac{TP}{TP+FN}$$

F-measure, it is also called F score, is the harmonic mean of precision and recall which is given by the equation:

$$f_{measure}=\frac{2^{\text{*}}PRE^{\text{*}}REC}{PRE+REC}$$

ROC stands for Receiver operating characteristic. It's created by plotting the True Positives rates versus False Positives rates. It is also exploited to evaluate the performance of classifiers as Area Under ROC.

## 3 Results and discussion

The dimensionality of the dataset was reduced by applying attribute selection before being passed on to KLR. The two evaluators selected were correlation and chi-squared. In Table 1 are presented the KLR performance evaluation results applied on the original data to comparison. These results are expressed on average, considering the 10 times that each test was repeated.

**Table 1:** Results achieved with 10-fold cross-validation.

	KLR	MetaLearner (correlation-KLR)	MetaLearner (chi-squared-KLR)
ACC (%) (st. dev.)	98.17 (8.17)	98.50 (7.53)	98.50 (7.53)
*κ* (st. dev.)	0.95 (0.20)	0.97 (0.14)	0.97 (0.16)
MAE (st. dev.)	0.02 (0.06)	0.01 (0.05)	0.01 (0.05)
Recall (st. dev.)	1	1	0.98 (0.11)
F-measure (st. dev.)	0.99 (0.06)	0.99 (0.07)	0.99 (0.06)
Area under ROC (st. dev.)	1	1	1

*Statistically different at significance level 0.05.

The results of metalearner correlation-KLR and metalearner chi-squared-KLR presented in Table 1 were achieved with 71 features. The obtained results validate the reduction procedure as do not present statistically significant differences. The prediction results of KLR presents ACC ≈ 98.17%, whereas metalearner correlation-KLR and metalearner chi-squared-KLR presents the same ACC ≈ 98.50%. Kappa coefficient results of the three methods indicate almost perfect agreement between the classification and the true value. Recall and Area under ROC are equal to 1 on the three methods, except in recall on metalearner chi-squared-KLR that achieved 0.98. F-measure results were the same for all methods, achieving 0.99.

**Figure 2:**
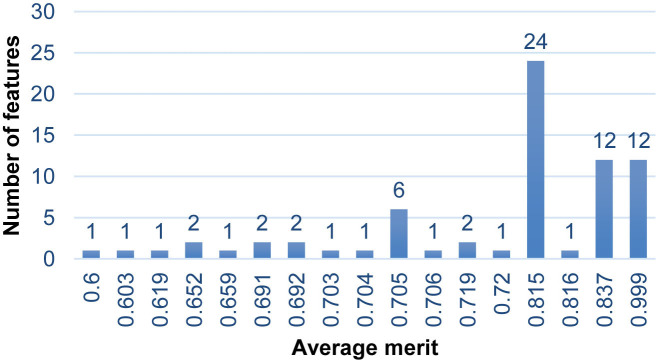
Average merit of information gain attribute selection after used metalearner-correlation-KLR with 10-fold cross-validation.

**Figure 3:**
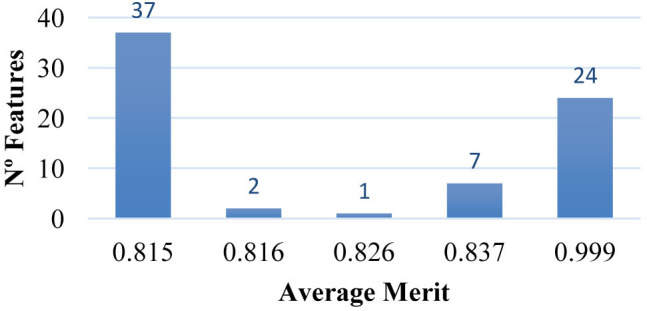
Average merit of information gain attribute selection after used metalearner-chi-squared KLR with 10-fold cross-validation.

After having found the reduced number of features without affecting the performance evaluation of the implemented classifier, the features retained by the two metalearner: correlation-KLR and chi-squared-KLR; were subjects to the information gain attribute evaluator. It allowed to determine the goodness of an attribute by measuring the class information gained as a result of adding it to the list of input attributes. The results of the average merit of information gain attribute selection after used metalearner-correlation-KLR are presented in Figure 2 and the results of the average merit of information gain attribute selection after used metalearner-chi-squared KLR are presented in Figure 3.

In Table 2 are presented the features with highest score obtained (0.999) and the respective gene name/protein reported in the literature. The respective *p*-values are also present.

**Table 2:** Features with highest average merit of information gain attribute selection after used metalearner correlation-KLR with 10-fold cross-validation.

Feature	Gene name (Protein)	*p*-value
39318_at	TCL1A (T cell leukemia/lymphoma 1A)	1.38057E-13
1389_at	MME (membrane metallo-endopeptidase)	8.81046E-06
31797_at	TBPL1 (TBP-like 1)	4.08536E-07
1456_s_at	IFI16 (Gamma-interferon-inducible protein)	1.67549E-06
37508_f_at	FUBP3 (Far upstream element-binding protein)	4.83968E-09
37988_at	CD79B (B-cell antigen receptor complex-associated protein beta chain)	6.735E-07
38242_at	SLP65 (B-cell linker protein)	5.73938E-06
32541_at	PPP3CC (protein phosphatase 3 (formerly 2B)	4.01276E-06
34168_at	DNTT (DNA deoxynucleotidyltransferase)	8.78887E-08
32315_at	RPS24 (ribosomal protein S24)	9.8389E-10
266_s_at	CD24 (Signal transducer CD24)	6.64401E-11
40701_at	USP13 (Ubiquitin carboxyl-terminal hydrolase)	8.47601E-07

**Table 3:** Features with highest average merit of information gain attribute selection after used metalearner chi-squared-KLR.

Feature	Gene name (Protein)	*p*-value
32872_at	TCF4 (Transcription factor 4)	9.4977E-05
36239_at	POU2AF1 (POU domain class 2-associating factor 1)	2.42789E-05
40505_at	UBE2L6 (Ubiquitin-conjugating enzyme E2L 6)	7.0848E-05
266_s_at	CD24 (Signal transducer CD24)	6.64401E-11
34168_at	DNTT (DNA deoxynucleotidyltransferase)	8.78887E-08
35164_at	WFS1 (Wolframin)	0.003024172
1389_at	MME (Neprilysin)	8.81046E-06
1456_s_at	IFI16 (Gamma-interferon-inducible protein 16)	1.67549E-06
39318_at	TCL1A (T cell leukemia/lymphoma 1A)	1.38057E-13
33154_at	PSMB4 (proteasome subunit beta 4)	4.26805E-06
37988_at	CD79B (CD79B antigen immunoglobulin-associated beta)	6.735E-07
32315_at	RPS24 (ribosomal protein S24)	9.8389E-10
33374_at	C2 (complement component 2)	0.000998743
32847_at	MYLK (Myosin light chain kinase, smooth muscle)	0.000681112
754_s_at	BCR (Breakpoint cluster region protein)	5.61522E-06
40701_at	USP13 (Ubiquitin carboxyl-terminal hydrolase)	8.47601E-07
32579_at	SMARCA4 (Transcription activator BRG1)	7.58503E-05
31797_at	TBPL1 (TBP-like 1)	4.08536E-07
35775_at	SMYD2 (*N*-lysine methyltransferase SMYD2)	8.45417E-06
31855_at	SRPX (Sushi repeat-containing protein SRPX)	1.76652E-06
37508_f_at	FUBP3 (Far upstream element-binding protein 3)	4.83968E-09
38242_at	SLP65 (B-cell linker protein)	5.73938E-06
34322_r_at	FAM3C (Protein FAM3C)	0.002052207
32541_at	PPP3CC (Serine/threonine-protein phosphatase 2B catalytic subunit gamma isoform)	4.01276E-06

As demonstrated below the genes that emerged from the information gain evaluator are correlated with the studied disease. TCL1A encodes T-cell leukemia/lymphoma protein 1A. This gene enhances the phosphorylation and activation of AKT1, AKT2 and AKT3. It enhances cell proliferation, promotes cell survival and stabilizes mitochondrial membrane potential [13], [14], [15]. Its expression is deregulated in chronic lymphocytic leukemia and most lymphomas [16]. According to Uniprot database, MME encode neprilysin protein and it is an important cell surface marker in the diagnostic of human ALL (Table 3).

TBPL1 encodes TATA box-binding protein-like protein 1. It is part of a specialized transcription system that mediates the transcription of most ribosomal proteins [17]. A recent study [18] demonstrated that the expression of IFI16, a member of the PYHIN protein family involved in apoptosis regulation and proliferation inhibition, is associated with clinical outcome in chronic lymphocytic leukemia.

According to Uniprot database, FUBP3 may play a role in activation of gene expression and may interact with single-stranded DNA from the far-upstream element (FUSE). Referring to Uniprot database, CD79B encodes B-cell antigen receptor complex-associated protein beta chain. It is required in cooperation with CD79A for initiation of the signal transduction cascade activated by the B-cell antigen receptor complex (BCR) [19]. A study [20] reports that CD79B is found in mature B blasts (B-ALL) that express membrane Ig as it is in normal and leukemia B lymphocytes. SLP65 or BLNK play functions as a central linker protein, regulating biological outcomes of B-cell development and function, and downstream of the BCR [21], [22]. PPP3CC plays an essential role in the transduction of intracellular Ca_2_^+^ – mediated signals [23].

According to the Atlas of Genetics and Cytogenetics in Oncology and Haematology database, DNTT/ BLNK is related to ALL [24]. RPS24 is required for maturation of 40S ribosomal subunits and pre-rRNA [25]. This gene was identify on the top list of 20 genes as precursor of B-ALL [26]. It has been identified and characterized an increased risk of developing leukemia [27]. CD24 modulates B-cell activation responses and may have a pivotal role in cell differentiation of different cell types [28]. USP13 is involved in various processes such autophagy and endoplasmic reticulum-associated degradation [29], [30].

## 4 Conclusions

In this work, we have applied metalearners to reduce the number of features in order to optimize the informative genes prioritization. Metalearner correlation-KLR and metalearner chi-squared-KLR provided the methods to reduce the number of features to 71, the minimal number conserving the optimal classifying potential. Using the information gain attribute evaluator, we were able to identify the most promising biomarkers for Leukemia, based on the highest average merit score. In this way, it was possible to gather 12 common genes to the two metalearner reduction results. Furthermore, based on literature and protein databases we were able to confirm that the metalearner results are, mostly, coincident with laboratory studies identifying the same genes involved in Leukemia. In conclusion, the used metalearners proved to be effective methods to optimize the informative gene discovery and therefore can be relevant to corroborate diagnostic and prognostic of time critical diseases like cancer.
